# Knowing the Consequences of Unprotected Sex with Seroconcordant Partner Is Associated with Increased Safer Sex Intentions among HIV-positive Men in Kathmandu, Nepal

**DOI:** 10.3329/jhpn.v29i3.7866

**Published:** 2011-06

**Authors:** Krishna C. Poudel, Kalpana Poudel Tandukar, Shinji Nakahara, Junko Yasuoka, Masamine Jimba

**Affiliations:** ^1^Department of Community and Global Health, Graduate School of Medicine, University of Tokyo, Tokyo, Japan; ^2^Waseda Institute for Advanced Studies, Waseda University, Tokyo, Japan; ^3^Department of Preventive Medicine, St. Marianna University School of Medicine, Kawasaki, Japan

**Keywords:** HIV infections, Intention, Knowledge, Knowledge, attitudes, practices, Perceptions, Safe sex, Sexual behaviour, Nepal

## Abstract

Unprotected sexual intercourse among HIV-positive people can adversely affect their own health by increasing their exposure to multiple strains of HIV-1 or other sexually transmitted infections (STIs). The study explored the relationship between knowledge of Nepalese HIV-positive men about the consequences of having unprotected sex with seroconcordant partners and their intention to practise safer sex with such partners. In total, 166 participants recruited conveniently in the Kathmandu Valley, Nepal, were interviewed. Each participant reported intention to practise safer sex with seroconcordant partners, know-ledge about the consequences of having unprotected sex with seroconcordant partners, perceived partner-related barriers to condom-use, belief that condoms interfere with sex, and condom-use self-efficacy. Of the 166 participants, 50.6% intended to practise safer sex every time they have sex with seroconcordant partners. Results of multiple logistic regression analysis showed that the participants who were aware of the possibility of HIV superinfection [adjusted odds ratio (AOR)=2.93, 95% confidence interval (CI) 1.16-7.34, p=0.022)] or that the presence of STIs in HIV-positive persons increases progression of HIV disease (AOR=2.80, 95% CI 1.08-7.26, p=0.033) were more likely to intend to practise safer sex with seroconcordant partners. Similarly, the participants who were employed or who had lower levels of belief that condoms interfere with sex were more likely to intend to practise safer sex. The findings suggest that improving the knowledge of HIV-positive persons about the consequences of having unprotected sex with seroconcordant partners might improve their intention to practise safer sex with such partners.

## INTRODUCTION

HIV-positive people sometimes engage in unprotected sexual intercourse with seroconcordant partners, regardless of whether partners are primary or non-primary (1-5). Such behaviour on the part of HIV-positive people might be considered a rational response as it does not spread HIV infection to negative partners.

However, unprotected sexual intercourse among HIV-positive seroconcordant partners can pose serious health risks for themselves. First, unprotected sexual intercourse between HIV-positive people can increase their risk of superinfection with other strains of HIV, including drug-resistant viral strains (6). Second, it can also increase the risk of their exposure to other sexually transmitted infections (STIs) that can accelerate their HIV disease. The presence of syphilis and other genital ulcer diseases can exacerbate HIV disease by increasing viral load in the blood and genital secretions and by decreasing CD4 cells (7-9).

Although there are serious risks associated with unprotected sex among HIV-positive seroconcordant partners, little is known about how the knowledge of HIV-positive people about such risks influences their intention to practise protected sex (safer sex). A qualitative study reported that several HIV-positive people who did not know the consequences of having unprotected sex with seroconcordant partners did not think that they were at risk of ill-health, despite their risky behaviours; such HIV-positive people had negative perceptions towards ‘safer sex’ practices with seroconcordant partners (10).

Health information has an important role in predicting health-related behaviours by changing perceptions of risk (11-13). As the Protection Motivation Theory suggests, information on health hazard stimulates a cognitive assessment of vulnerability to the negative event, along with assessments of the severity and efficacy of the recommended precautionary actions (14). According to Rogers, such assessment acts as a mediator of the persuasive effects of the message by motivating individuals to protect themselves (14). From this perspective, one's risk-taking is likely shaped by a lack of knowledge on the health hazards of own risky behaviours, such as the possibility of HIV superinfection or HIV disease progression as a result of having unprotected sex among seroconcordant HIV-positive people.

Previously, only a few studies have explored the factors associated with unprotected sex among HIV-positive seroconcordant parters in developed countries. One study in France reported that HIV-positive men who have sex with men (MSM) who reported more than four casual partners or who had unprotected sex with non-regular partners in the previous year were more likely to practise unprotected sex with their regular seroconcordant partners (15). Another study in the United States revealed that HIV-positive MSM who believed that superinfection damaged their health were less likely to practise unprotected anal sex than those who did not believe it (16). However, collection of data in the later study was done before the scientific confirmation of HIV superinfection in humans.

Nepal has been facing a serious HIV/AIDS epidemic, with approximately 70,000 HIV-positive people at the end of 2007 (17). Although the national HIV prevalence among 15-49 years old population is about 0.5%, the HIV prevalence is reportedly over 5% among injecting drug-users (IDUs), female sex workers (FSWs), and migrant workers to India (17,18). Previous HIV-prevention efforts in Nepal were mainly directed at high-risk groups, such as IDUs, FSWs and their clients, mobile populations, MSM, and prisoners without considering their HIV status (19). The latest national HIV/AIDS strategy of Nepal (2006-2011) identified HIV-positive people as a target group for HIV-prevention interventions (20). Better understanding of sexual behaviours of HIV-positive people or their intention to practise such behaviours can help design effective interventions.

Previously, we assessed unsafe sexual behaviours of Nepalese HIV-positive men and their knowledge about the consequences of unsafe sex (21). In this study, 46% of 123 participants who had sex in the past six months did not always use condoms with seroconcordant, serodiscordant, or with sero-unknown partners. However, in that study, we could not explore the relationship between the knowledge of participants about the consequences of having unprotected sex with seroconcordant partners and their sexual behaviours with such partners as only 23 of the 123 sexually-active participants had sex with known HIV-positive partners.

Therefore, this study aimed at exploring the relationship between knowledge about the consequences of having unprotected sex with seroconcordant partners and the intention to practise safer sex with such partners among HIV-positive men in the Kathmandu Valley, Nepal. We hypothesized that knowledge of HIV-positive men about the consequences of having unprotected sex with seroconcordant partners would promote their intention to practise safer sex with such partners, independently of other confounding factors.

## MATERIALS AND METHODS

### Study design and sample

We conducted the study in the Kathmandu Valley, Nepal, with 167 HIV-positive men described elsewhere (21). The inclusion criteria of the participants were: age 18-50 years, self-reported diagnosis of HIV infection at least three months before the date of interview, and willing to participate voluntarily in the study.

We selected the participants using a snowball sampling method as the detailed database of HIV-positive people was not available to select them randomly. For this, we identified the initial participants through the members of the local organizations or the networks of HIV-positive people in the Kathmandu Valley. The Kathmandu Valley consists of three (Kathmandu, Lalitpur, and Bhaktapur) of 75 districts in Nepal, with an estimated population of 1.9 million in 2006. In the Kathmandu Valley, the HIV prevalence was 35% among IDUs in 2007, 3% among MSM in 2007, and 1.4% among FSWs in 2006 (22).

### Collection of data

A structured pretested questionnaire in Nepali language was used for conducting interviews during March-June 2006. We hired four local interviewers who were also HIV-positive men and gave them a one-day training. Using the information-sheet, these interviewers first informed all the participants individually about the study and requested their voluntary participation in it. Then, the interviewers conducted face-to-face interviews in a private place. Each interview took about 30-40 minutes.

To improve confidentiality, the interviewers re-assured each participant that they would use codes in all the records rather than their names. Assurances of confidentiality have been shown to enhance the participants’ candid reporting of sensitive behaviours (23).

### Measures

Measures included sociodemographic and health characteristics, intention to practise safer sex with seroconcordant partners, knowledge about the consequences of having unprotected sex with seroconcordant partners, availability of condoms, embarrassment to buy or ask for condoms, perceived partner-related barriers, belief that condom interferes with sex, and condom-use self-efficacy.

We measured intention of the participants to practise safer sex with seroconcordant partners with the question ‘Do you intend to use condoms with HIV-positive sex partners?’ Similar to a previous study (24), the participants responded to this question with: 1 ‘Never’, 2 ‘Sometimes’, 3 ‘Most of the time’, and 4 ‘Every time’. We dichotomized these responses because of the skewed score (median=4); we collapsed ‘Never’, ‘Sometimes’, and ‘Most of the time’. After dichotomatization, a high desirable score (favouring condom-use) was coded 1 and a low undesirable score 0.

Knowledge of the participants about the possible consequences of having unprotected sex using three true-false questions was measured. Questions included the participants’ knowledge about that ‘HIV-positive persons can become sicker if they have unprotected sex with a person who has HIV or other STIs’, or that ‘the presence of STIs in an HIV-positive person increases the HIV disease progression’, or that ‘HIV superinfection is possible’. The questions were developed based on previous studies by Colfax *et al.* (16) and Wingood *et al.* (25).

We measured ‘perceived partner-related barriers to condom use’ using a six-item scale (α=0.90). We measured the participants’ ‘belief that condoms interfere with sex’ using an eight-item scale (α=0.87). These measures were adopted from a previous study by Wingood *et al*. (25). Items of both the scales were rated on a four-point response scale from 1 ‘Strongly disagree’ to 4 ‘Strongly agree’. We averaged across the items of each scale to form composite measures; the median score of each scale was 2.33 and 2.50 respectively. We then categorized the score as high or low levels for analysis. The low level included median scores or below-median scores, and the high level included scores above the median.

The confidence of the participants in their ability to use condoms properly (condom-use self-efficacy) was measured using an eight-item scale (α=0.72). A sample question included ‘Unroll a condom down correctly on the first try’. The original scale had nine items (25). However, we deleted one item—‘Use spermicide or lubricant with a condom’, as the practice is not common in Nepal. Each item had a response scale ranging from 0 ‘Not at all confident’ to 2 ‘Very confident’. The median score of condom-use self-efficacy was 1.25. For analysis, we categorized the score as high or low levels. The high level included scores above the median and the low level included scores below or at the median score.

We measured the availability of condom and embarrassment to buy or ask for condoms as ‘Most of the time we do not have condoms when we need one’ and ‘I would be embarrassed to buy or ask for condoms’ (25) respectively. The participants rated these items on a four-point scale, ranging from (a) strongly disagree to (d) strongly agree. For analysis, these responses were coded as ‘disagree’ or ‘agree’ to reduce the influence of random error.

### Analysis of data

Of the 167 participants who completed the survey, the responses of 166 participants were included in our analysis. We excluded one participant from analysis as the time of HIV diagnosis of this person was not known. First, we analyzed sociodemographic variables using descriptive statistics; we reported mean and standard deviation (SD) or median and inter-quartile range (IQR) as appropriate. We then examined bivariate associations between each independent variable and participants’ intention to practise safer sex with seroconcordant partners. Finally, we examined the associations between the participant's knowledge about the consequences of having unprotected sex with seroconcordant partners and their intention to practise safer sex with such partners using multiple logistic regression analysis. In multivariable analysis, we included sociodemographic characteristics and psychosocial mediators, such as condom-use self-efficacy or belief that condoms interfere with sex as potential confounding factors despite their statistical significance in bivariate analysis. These variables were selected as these are known to be predictors of condom-use or intentions to use condom (26,27). The multicollinearity was performed to assess possible collinearity among covariates; the variance inflation factor of all variables was less than 2.0. We performed all the analyses using the SPSS software (version 15.0) (SPSS Inc, Chicago, USA), with statistical significance set at p<0.05.

### Ethical approval

The Nepal Health Research Council approved the procedures of the study. All the participants agreed to participate in the study and signed the informed consent form.

## RESULTS

### General characteristics

The mean age of the 166 participants was 30.5 (SD=5.7) years. Of them, 46% were currently married, 66% had above primary-level education, and 69% were employed (Table 1). The median period for the participants since testing HIV-positive was 25 months (IQR=12-48). Of the participants, 16% were on antiretroviral treatment at the time of data collection. One hundred sixty-three (98%) participants identified themselves as heterosexual while the other three identified themselves as bisexual. Of the participants, 134 (81%) believed that they contracted HIV by sharing injection equipment, 27 (16%) by having unsafe sex, and five were not sure if it was by sharing injection equipment or by having unsafe sex.

**Table 1.** Bivariate logistic regression analysis of predictors of intention to practise safer sex in seroconcordant relationship among HIV-positive men in the Kathmandu Valley, Nepal

Variable	Intention to practise safer sex				OR	95% CI	p value
	High (n=84)		Low (n=82)				
	No.	%	No.	%			
Age (years)							
31-45	39	50	39	50	0.95	0.51-1.75	0.884
19-30	45	51	43	49			
Marital status							
Currently married	43	57	33	43	1.55	0.84-2.88	0.157
Currently non-married	41	46	49	54			
Education*							
Above primary	62	57	47	43	2.12	1.05-4.27	0.033
Primary or less	18	38	29	62			
Employment†							
Yes	68	59	47	41	3.27	1.60-6.68	0.001
No	15	31	34	69			
Months since testing HIV⩲							
25 or less	41	49	43	51	0.86	0.47-1.59	0.640
26 or more	43	52	39	48			
On antiretroviral treatment							
Yes	18	67	9	33	2.21	0.93-5.26	0.068
No	66	47	73	53			
Perceived partner-related barriers†							
Low	52	57	40	43	1.71	0.92-3.20	0.157
High	31	43	41	57			
Belief that condom interferes with sex†							
Low	57	65	31	35	3.53	1.85-6.73	<0.001
High	26	34	50	66			
Condom-use self-efficacy‡							
High	42	54	36	46	1.25	0.67-2.30	0.475
Low	42	48	45	52			
I would be embarrassed—buy or ask for condoms¶							
Disagree	70	61	44	39	4.40	2.10-9.21	<0.001
Agree	13	27	36	73			
Most of the time we do not have condoms when we need one†							
Disagree	41	49	43	51	0.86	0.46-1.59	0.637
Agree	42	53	38	47			
HIV-positive person can become sicker if they have unprotected sex with another HIV-positive person							
True	66	60	44	40	3.16	1.60-6.24	0.001
False	18	32	38	68			
Presence of STIs in a HIV-positive person increases HIV disease progression							
True	64	62	39	38	3.52	1.81-6.84	<0.001
False	20	32	43	68			
Knowledge about HIV superinfection							
Yes	46	77	14	23	5.88	2.86-12.05	<0.001
No	38	36	68	64			

*Ten participants did not respond to this question;

†Two participants did not respond to this question;

‡Two participants did not respond to this question;

¶Three participants did not respond to this question;

CI=Confidence interval;

HIV=Human immunodeficiency virus;

OR=Odds ratio;

STIs=Sexually transmitted infections

### Intention to practise safer sex

Of the HIV-positive men in the study, 51% (n=84) intended to practise safer sex every time they have sex with HIV-positive partners. Forty-nine (29%) intended to practise safer sex most of the time they have sex, and 11 (7%) intended to practise safer sex sometimes when they have sex with HIV-positive partners. The remaining 22 (13%) did not intend to practise safer sex in their sexual intercourse with HIV-positive partners.

### Predictors of intention to practise safer sex

In bivariate analysis, the variables that were significantly associated with a positive intention to practise safer sex with seroconcordant partners included: having above primary-level education (p=0.033), having employment status (p=0.001), having low level of belief that condoms interfere with sex (p<0.001), having disagreement that they would be embarrassed to buy or ask for condoms (p<0.001), having the knowledge that ‘HIV-positive persons can become sicker if they have unprotected sex with a person who has HIV or other STIs’ (p=0.001), ‘the presence of STIs in HIV-positive persons increases the HIV disease progression’ (p<0.001), or ‘HIV superinfection is possible’ (p<0.001) (Table 1).

In multiple logistic regression analysis, the participants who were employed or who had low levels of belief that condoms interfere with sex were more likely to intend to practise safer sex with seroconcordant partners than those who were not employed (AOR)=3.50, 95% CI 1.33-9.20, p=0.011) or those who had high levels of belief that condoms interfere with sex (AOR=3.10, 95% CI 1.18-8.15, p=0.021) (Table 2). Similarly, the participants who knew that ‘the presence of STIs in HIV-positive persons increases the HIV disease progression’ (AOR=2.80, 95% CI 1.08-7.26, p=0.033) or ‘HIV superinfection is possible’ (AOR=2.93, 95% CI 1.16-7.34, p=0.022) were more likely to intend to practise safer sex with seroconcordant partners. The currently-married participants were more likely to intend to practise safer sex with seroconcordant partners than the currently-non-married participants. Although the difference was not statistically significant, the adjusted OR increased (AOR=2.37, 95% CI 0.98-5.72).

**Table 2.** Multivariate logistic regression analysis of predictors of intention to practise safer sex in seroconcordant relationship among HIV-positive men in the Kathmandu Valley, Nepal

Variable	AOR*	95% CI	p value
Age (years)			
31-45	0.60	0.24-1.47	0.266
19-30			
Marital status			
Currently married	2.37	0.98-5.72	0.053
Currently non-married			
Education			
Above primary	1.73	0.68-4.35	0.244
Primary or less			
Employment			
Yes	3.50	1.33-9.20	0.011
No			
Months since testing HIV-positive			
25 or less	1.59	0.63-4.00	0.318
26 or more			
On antiretroviral treatment			
Yes	1.90	0.56-6.38	0.299
No			
Perceived partner-related barriers			
Low	0.97	0.35-2.68	0.962
High			
Belief that condom interferes with sex			
Low	3.10	1.18-8.15	0.021
High			
Condom-use self-efficacy			
High	0.50	0.21-1.18	0.115
Low			
I would be embarrassed to buy or ask for condoms			
Disagree	2.54	0.90-7.17	0.076
Agree			
Most of the time we do not have condoms when we need one			
Disagree	0.65	0.25-1.69	0.382
Agree			
HIV-positive person can become sicker if they haveunprotected sex with another HIV-positive person			
True	1.23	0.46-3.26	0.667
False			
Presence of STIs in a HIV-positive person increasesthe HIV disease progression			
True	2.80	1.08-7.26	0.033
False			
Knowledge about HIV-superinfection			
Yes	2.93	1.16-7.34	0.022
No			

*152 participants were included in the multiple logistic regression analysis;

AOR=Adjusted odds ratio;

CI=Confidence interval;

HIV=Human immunodeficiency virus;

STIs=Sexually transmitted infections

## DISCUSSION

The results of the study revealed that HIV-positive Nepalese men are likely to intend to practise safer sex with seroconcordant partners when they know about the consequences of having unprotected sex with such partners, particularly about HIV superinfection or that the presence of STIs in HIV-positive persons increases the HIV disease progression. Our results have potential implications for counselling HIV-positive people about their sexual behaviours. Although we measured the intention of the participants to practise safer sex, our results suggest that safer sex-related messages to HIV-positive people should include the concern about HIV superinfection and the role of STIs to exacerbate HIV disease and suggest that they use condoms with seroconcordant partners to prevent the possibility of HIV superinfection and other STIs.

Our findings are consistent with the view of the Protection Motivation Theory (14,28). As this theory suggests, it seems that the knowledge of the participants about the consequences of practising unprotected sex with seroconcordant partners increased the heightened concerns about their own health to practise unprotected sex with such partners. A qualitative study in Kathmandu also reported that HIV-positive people who did not know about HIV superinfection did not think that they were at risk of any serious health conditions despite their risky behaviours (10).

The participants with low levels of belief that condoms interfere with sex were more likely to intend to practise safer sex with seroconcordant partners. This result is consistent with results of previous studies, showing a relationship between low levels of perceived sexual pleasure with condoms and risky behaviours (29,30). The implication of our findings is that HIV-prevention interventions targeting HIV-positive people need to discover their perceptions about using condoms. The more positive their attitudes can be made towards using condoms, the greater is the likelihood that HIV-positive people will engage in safer sex with seroconcordant partners.

Compared to the currently-non-married participants, the currently-married participants tended to intend to practise safer sex with seroconcordant partners, although the association was not statistically significant; the OR increases after taking into account the other variables in multivariable analysis. Our result is in line with the results of a previous study in Nepal, which reported that married male participants were less likely to engage in risky sexual behaviours than unmarried male participants (31).

The participants who were employed were more likely to intend to practise safer sex with seroconcordant partners compared to those who were not employed. Although further studies may explore the possible mechanisms of this relationship, it is possible that the employed participants have an increased access to services and information because of their income than those of the unemployed participants. Our results suggest that HIV-prevention interventions should be tailored, particularly for those HIV-positive people who do not have any jobs.

### Limitations

This study has some limitations. First, the outcome variable of the study was intention to practise safer sex with seroconcordant partners and not the practice itself. In our study, we measured sexual behaviours of the participants (21). However, we could not explore the relationship between the knowledge of the participants about the consequences of having unprotected sex with seroconcordant partners and their sexual behaviours with such partners due to the small sample-size of participants who had sex with known HIV-positive partners. Previous studies have shown positive correlations between the intention and the practice of condom-use; a metaanalysis showed an average of 0.43 correlations between intention to use a condom and actual condom-use (26). With the increasing HIV testing facilities, it is expected that more HIV-positive people will be aware about the HIV-positive status of their partners. An implication of our results for future research is, therefore, to examine the influence of the knowledge of HIV-positive people, including HIV-positive women, about the consequences of having unprotected sex with seroconcordant partners on their actual behaviour regarding condom-use with such partners using a longitudinal design. Second, the responses of the study participants might have been influenced by the social desirability bias as all the measures were self-reported. However, interviews were conducted in a confidential setting, and the interviewers assured the participants that their responses would remain confidential. Third, our measurement of intention to practise safer sex was not specific to the type of partners. Finally, our participants might not represent all the HIV-positive men in the study area because our participants were selected using the networks of HIV-positive people in the Kathmandu Valley. HIV-positive men who were not members of such networks were not included in our study. As most of our participants identified themselves as heterosexual, it seems that only a few HIV-positive MSM were included in this study.

### Conclusion

The findings of our study revealed a positive relationship between knowledge of the Nepalese HIV-positive men about the consequences of having unprotected sex with seroconcordant partners and their intention to practise safer sex with such partners. Although longitudinal studies are necessary to draw decisive conclusions, our findings suggest that HIV-prevention interventions among HIV-positive people should aim to improve their knowledge about the consequences of having unprotected sex with seroconcordant partners on them. As our results highlight, the improved knowledge of HIV-positive people about the consequences of having unprotected sex with seroconcordant partners might improve their intention to practise safer sex with such partners.

## ACKNOWLEDGEMENTS

The authors thank all the study participants for their participation. They also thank other people who assisted them in recruiting the participants and managing the appropriate places for interviews.
